# Emergency department utilization before and during the COVID-19 pandemic among individuals with sickle cell disease

**DOI:** 10.1186/s12873-024-01043-5

**Published:** 2024-07-29

**Authors:** Brandon K. Attell, Allison P. Plaxco, Mei Zhou, Jhaqueline Valle, Sarah L. Reeves, Pooja N. Patel, Krista Latta, Matthew P. Smeltzer, Angela B. Snyder

**Affiliations:** 1 Georgia Health Policy Center, Georgia State University, 55 Park Place NE, Atlanta, GA 30303 USA; 2 Division of Epidemiology, Biostatistics, and Environmental Health, School of Public Health, University of Memphis, 222 Robison Hall, Memphis, TN 38152 USA; 3 Tracking California Program, Public Health Institute, 555 12th Street, Suite 600, Oakland, CA 94607 USA; 4 Susan B. Meister Child Health Evaluation and Research Center, Medical School, Department of Pediatrics, University of Michigan, 2800 Plymouth Road, Ann Arbor, MI 48109 USA; 5 Department of Epidemiology, School of Public Health, University of Michigan, 1415, Washington Heights, Ann Arbor, MI 48109 USA

**Keywords:** Sickle cell disease, COVID-19, Emergency department, Acute care, Healthcare utilization, Trends

## Abstract

**Background:**

The emergency department (ED) is a vital source of healthcare for individuals living with sickle cell disease (SCD). Prior research indicates that during the COVID-19 pandemic some individuals with SCD avoided the ED for fear of acquiring COVID-19 or delayed visiting the ED by self-management of symptoms or pain crisis at home. The purpose of the current study was to understand ED utilization rates before and during the pandemic among individuals living with SCD.

**Methods:**

We conducted a retrospective cohort study using population-based SCD surveillance systems in California, Georgia, Michigan, and Tennessee to assess the impact of the pandemic on ED utilization among people with SCD by (1) analyzing trends in monthly ED utilization from January 2019 - December 2020, with specific attention given to immediate changes at the onset of the pandemic; and (2) calculating changes in the volume of utilization by comparing the total ED visits made from March - December 2020 to the same period in 2019, both overall and by demographic characteristics.

**Results:**

Across all states, a decline in ED utilization during the onset of the pandemic was seen, with the largest decline seen in those under age 10. By December 2020, utilization rates were higher than their lowest observed month of April 2020, but had not fully returned to pre-COVID levels. During the pandemic, ED visits in each state decreased by as much as 25%, and the number of people with any ED utilization decreased by as much as 26%.

**Conclusions:**

This study confirms and extends the existing literature related to the impact of the pandemic on healthcare utilization patterns in the US, in a unique population with increased healthcare needs.

**Supplementary Information:**

The online version contains supplementary material available at 10.1186/s12873-024-01043-5.

## Background

Sickle cell disease (SCD) is a rare disease, affecting approximately 100,000 people in the United States (U.S.). Major complications of SCD include hemolytic anemia, infection, debilitating pain, stroke, and progressive organ damage [1, 2]. In the U.S. approximately 223,000 yearly emergency department (ED) visits occur on average among individuals with SCD, with 22% of those visits representing a follow-up from a prior episode of care [3]. One of the most common symptoms of SCD is vaso-occlusive crisis (VOC), which causes oxygen deprivation to body tissue caused by sickled red blood cells. VOC and other acute exacerbations of SCD regularly result in acute pain episodes that may require pain management procedures commonly administered in the ED setting, [4, 5] leading to frequent ED utilization in the SCD population, especially for severe pain [3, 6]. Additionally, the identification and treatment of SCD-related morbidities, such as acute chest syndrome and emergency blood transfusions, are often carried out in the ED setting [7]. Moreover, patients facing access to care issues or living in areas with no SCD specialists may rely on the ED as a source of regular treatment for SCD [8]. 

Individuals living with SCD commonly have impaired immune systems and underlying cardiopulmonary comorbidities, placing them at high risk for experiencing adverse outcomes such as hospitalization and mortality when they acquire SARS-CoV-2, the virus that causes COVID-19. While recent studies suggest that individuals with SCD who have acquired COVID-19 usually have a mild to moderate disease course, their risk for hospitalization is as high as 15 times that of individuals without SCD [9, 10]. Moreover, those with SCD have a higher risk of COVID-19 related death compared to people without SCD, and nearly 10% of deaths among the SCD population during 2020 were associated with COVID-19 [9, 11–13]. 

While the ED is an important source of care for individuals living with SCD, very little is known about the extent to which the COVID-19 pandemic impacted ED utilization for this population. There are several converging reasons to expect some level of change in ED utilization. Early social distancing guidelines urged individuals with SCD to stay at home except under emergency situations, [14] and results from qualitative studies indicate that some individuals with SCD actively avoided the ED in fear of acquiring COVID-19 in the healthcare setting [15]. Given the pandemic, individuals with SCD may have avoided or delayed visiting the ED by self-management of symptoms or pain crisis at home [16, 17]. This is especially true as healthcare systems rapidly increased the utilization of telehealth services throughout the pandemic, in which patients with SCD may have requested telehealth care instead of presenting to the ED [18–20]. However, acquisition of COVID-19 or adverse effects due to delay in preventative care may have resulted in increased ED utilization throughout the pandemic.

Given the limited understanding of ED utilization during the COVID-19 pandemic, the current study aims to describe the volume and patterns of ED use among individuals living with SCD. Using Medicaid data from four U.S. states with population-based SCD surveillance, we contribute to the understanding of the impact of the pandemic on acute care utilization in two ways. First, we describe trends in the rate of monthly ED utilization from January 2019 through December 2020, with specific attention given to immediate changes in utilization brought about by the onset of the pandemic. Second, we calculate changes in the volume of ED utilization by comparing the total ED visits made during March through December 2020 to the same period in 2019, both overall and stratified by demographic characteristics.

## Methods

### Data sources and study population

The data for this study come from the California, Georgia, Michigan, and Tennessee Sickle Cell Data Collection (SCDC) programs. Each state participating in SCDC collects, merges, and deduplicates data from various sources, including newborn screening records, administrative datasets such as hospital discharge data and Medicaid claims data, death records, and clinical reports to estimate the annual prevalence of SCD. Individuals with SCD in each state were identified by one of the following criteria using validated case definitions: (1) identification of SCD status through a state newborn screening program and/or other clinical laboratory data sufficient to confirm SCD; (2) other cases without laboratory confirmation of SCD met the SCDC probable case definition within linked administrative datasets of three or more SCD-coded encounters during any 5-year period. Complete information regarding case definitions and the SCDC program have been previously described [21–24]. After identifying individuals with SCD in each state, we limited our study cohort to those with at least 18 months of Medicaid enrollment during the 24-month period beginning in January 2019 (start of the study window) and ending in December 2020 (conclusion of the study window). All individuals were included regardless of age.

### Emergency department utilization

The primary exposure of interest was ED utilization from January 2019 through December 2020. In California and Georgia, ED utilization data were obtained by linking each state’s study cohort with statewide hospital discharge records. In Michigan, ED utilization was collected using Medicaid claims among those identified in the study cohort. In Tennessee, Medicaid claims supplemented with hospital discharge records were used to identify ED utilization. Across all states, only ED visits that did not result in admission to the hospital were included, i.e., only “ED treat and release” visits were included. ED utilization was operationalized using two separate definitions: first, as the total number of ED visits across all individuals in the cohort; and second, as the total number of individuals in each state’s cohort with one or more ED visits. Therefore, the former represents a visit-level utilization measure while the latter represents a person-level utilization measure. The hospital discharge data and Medicaid claims data used in this study captured all ED visits incurred in the study sample. Such reliance on administrative data sources resulted in no missing data for the ED utilization measures.

### Analyses

The first goal of our analysis was to describe changes in the monthly trend of ED utilization before and during the COVID-19 pandemic. For the purposes of the descriptive trend analysis, the before COVID period was defined a priori as the 14 months spanning January 2019 through February 2020 and the during COVID period was defined a priori as the ten months spanning March 2020 through December 2020. For both the visit-level and person-level measures, we describe the monthly trends in utilization before and during the COVID-19 pandemic as well as the immediate change in utilization during the first two months of the pandemic. To better understand recovery of utilization following the pandemic, we also describe the utilization level in December 2020 (the last study month) relative to the level of utilization in February 2020 (the month immediately preceding the start of the pandemic). A supplemental interrupted time series analysis was conducted to determine if the observed trends were robust to seasonal variation in ED utilization (see Supplemental Material 1).

The second goal of our analysis was to examine changes in ED utilization, both overall and by demographic characteristics. We calculated percentage changes in the visit- and person-level ED utilization measures, comparing change in utilization between March 2020 through December 2020 to the corresponding time period in 2019. Percentage change was calculated for the overall cohort as well as by age, sex, and race/ethnicity. Age in years was calculated at the start of the study (January 2019) and subsequently stratified into ten-year age groups (for example, 0–9 years old, 10–19 years old, etc.) and top coded at age 60+. Sex was categorized by those who were male or female. In California and Michigan, race/ethnicity was captured as Black, non-Hispanic; White, non-Hispanic; other or unknown race, non-Hispanic; and Hispanic, regardless of race. In Tennessee, race/ethnicity was captured as Black, non-Hispanic; White, non-Hispanic; and other or unknown race/ethnicity. Race/ethnicity data was not available for Georgia.

Demographic characteristics and all ED utilization data were extracted from each state’s SCDC surveillance system using SAS Version 9.4 [25]. All analyses were conducted using R version 4.2.2 [26]. Visualizations were produced using the ggplot2 package [27]. 

## Results

Demographic characteristics of the study cohorts from each state are presented in Table 1. The largest number of individuals with SCD were from Georgia (*N* = 4,524), followed by California (*N* = 2,176), Michigan (*N* = 2,168), and Tennessee (*N* = 1,454). Across all states, younger individuals comprised the greatest percentage of the study cohort, with more than half of the individuals in each state being less than 30 years of age at the beginning of the study. Across all states there was a greater percentage of women than men, with women consistently comprising more than half of the sample in each state. Regarding race and ethnicity, in each state more than.

**Table 1:** Demographic characteristics of study cohorts

	California		Georgia		Michigan		Tennessee	
**Total individuals**	2,176		4,524		2,168		1,454	
	*N*	%	*N*	%	*N*	%	*N*	%
**Age**								
0–9	439	20.17%	1098	24.27%	421	19.42%	370	25.45%
10–19	453	20.82%	1253	27.70%	391	18.04%	360	24.76%
20–29	458	21.05%	715	15.80%	439	20.25%	254	17.47%
30–39	392	18.01%	651	14.39%	386	17.80%	234	16.09%
40–49	207	9.51%	371	8.20%	261	12.04%	115	7.91%
50–59	175	8.04%	247	5.46%	168	7.75%	86	5.91%
60+	52	2.39%	189	4.18%	102	4.70%	35	2.41%
**Sex**								
Male	1013	46.55%	1945	42.99%	873	40.27%	585	40.23%
Female	1163	53.45%	2579	57.01%	1295	59.73%	869	59.77%
**Race / Ethnicity**								
Black, non-Hispanic	1816	83.46%			1953	90.08%	1375	94.57%
White, non-Hispanic	29	1.33%			87	4.01%	27	1.86%
Other or Unknown Race, non-Hispanic	56	7.17%			105	4.84%	-	-
Hispanic, regardless of race	175	8.04%			23	1.06%	-	-
Other or Unknown Race/Ethnicity	-	-			-	-	52	3.58%

*Note* race/ethnicity data unavailable for Georgia. Dashes represent race/ethnicity data incongruent with other states

80% of the individuals included were Black, non-Hispanic. There were comparatively fewer White, non-Hispanic individuals (less than 5% across all states) and Hispanic individuals. (1% in Michigan and 8% in California). Complete race and ethnicity data was unknown for approximately 4–7% of the cohorts across states (excluding Georgia, where race/ethnicity data was unavailable).

**Fig. 1:**
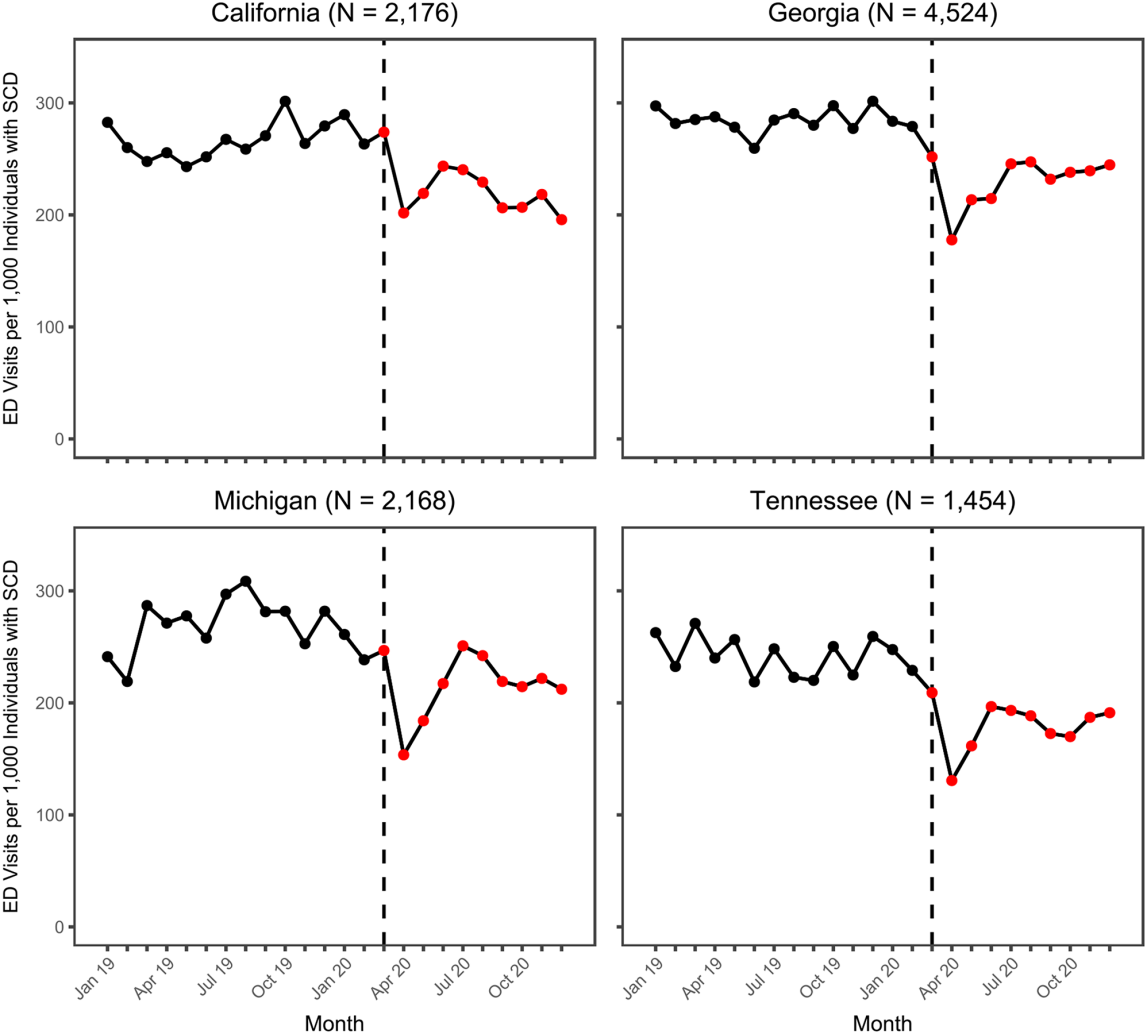
Monthly trends in emergency department visits per 1,000 Individuals with sickle cell disease. Black and red points represent the observed rate during the pre-pandemic and pandemic periods, respectively. The dashed black line represents March 2020, the start of the pandemic months

Results of the descriptive trend analysis for the number of ED visits across all individuals with SCD are displayed in Fig. 1. Prior to the start of the COVID-19 pandemic, in California, Georgia, and Michigan, rates of ED visits per 1,000 individuals with SCD generally tended to increase over time while in Tennessee there appeared to be more month-over-month fluctuation in the pre-pandemic utilization, with no strongly exhibited upward or downward trend. At the start of the pandemic, comparing March 2020 to February 2020, ED utilization slightly increased in California and Michigan (4% change in each state) and slightly decreased in Georgia and Tennessee (10% and 9% decrease, respectively). In all four states, ED utilization sharply dropped in April 2020, decreasing by 26% in California, 29% in Georgia, 37% in Tennessee, and 38% in Michigan. Except for California, monthly ED utilization in each state generally trended toward pre-pandemic levels following the lowest utilization level observed in April 2020. However, by December 2020 rates had not fully reached their pre-pandemic level. For example, in Michigan and Georgia, December 2020 utilization was 11% and 12% lower (respectively) than utilization in February 2020. Additional analyses indicated that these trends remained the same even after accounting for seasonal variation in ED utilization (see Supplemental Material 1).

**Fig. 2:**
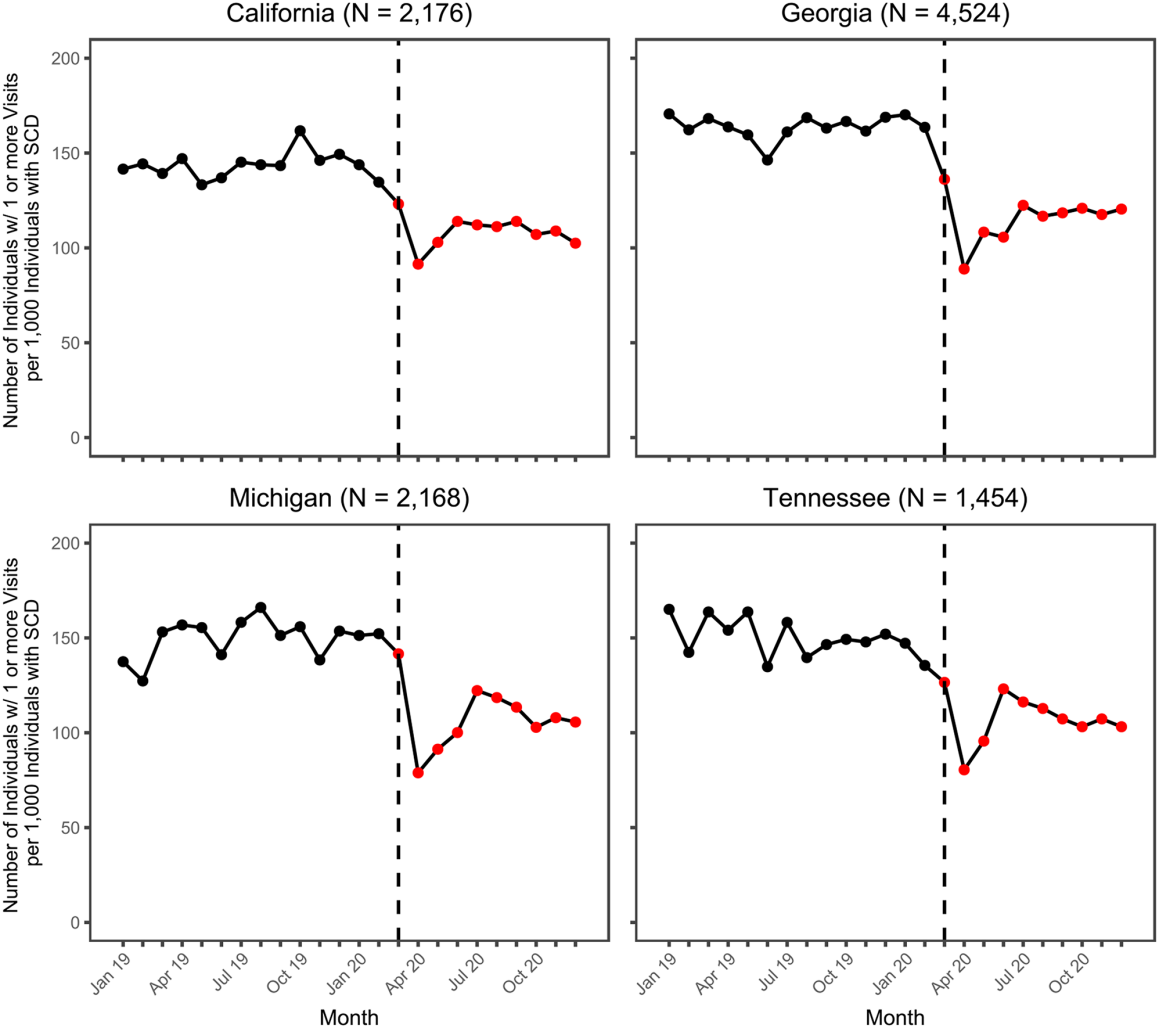
Monthly trends in the number of individuals with $\:\ge\:$1 ED Visits, per 1,000 with SCD. Black and red points represent the observed rate during the pre-pandemic and pandemic periods, respectively. The dashed black line represents March 2020, the start of the pandemic months

Results of the descriptive trend analysis for the number of individuals with one or more ED visits are displayed in Fig. 2. Prior to the start of the COVID-19 pandemic, person-level utilization slightly decreased in California and more clearly decreased in Tennessee. In Georgia, pre-pandemic person-level utilization generally remained the same despite some month-over-month fluctuation, while in Michigan pre-pandemic utilization slightly increased over time. At the start of the pandemic, comparing March 2020 to February 2020, person-level utilization rates.

Additional analyses indicated that these trends remained the same even after accounting for seasonal variation in ED utilization (see Supplemental Material 1).

Changes in the volume of total ED visits before and during the COVID-19 pandemic are displayed in Table 2. Across all states, the total number of ED visits across all individuals decreased between 15% (California) and 25% (Tennessee) during the pandemic. The greatest decrease in total visit volume was consistently seen for infants and young children aged 0–9 years, where the drop in utilization decreased between 54% in Michigan and as much as 61% in California. In California and Georgia, individuals aged 60 years and older experienced a decrease in total visit volume by nearly 40% during the pandemic. In California, Michigan, and Tennessee, similar declines in utilization were exhibited between men and women; in Georgia, men experienced a greater decline (24%) compared to women (16%). Regarding race and ethnicity, in Michigan and Tennessee Black individuals experienced a decrease in utilization by approximately 24% and in California this decline was approximately 15%. White individuals also experienced a decline in utilization (between 10% in Michigan and up to 51% in California),

but comparison between states is limited due to differences in the population size of White individuals with SCD. Total ED visits decreased by 15% for Hispanic individuals in California and 23% in Michigan.

Changes in the number of individuals with any ED utilization (1 or more visits) before and during the COVID-19 pandemic are displayed in Table 3. In California, Georgia, and Tennessee, the number of individuals with any ED utilization decreased by approximately 25% during the pandemic (22% decrease in Michigan). The greatest decline in utilization was consistently seen for infants and young children aged 0–9 years, where the number of individuals with any ED utilization dropped between 37% (in Michigan) and 54% (in California). Across all states, both men and women experienced a decline in the number of individuals with any utilization, although the decline was consistently greater in each state for men compared to women. This difference was most pronounced in Michigan, where women experienced a decline of approximately 20% and men experienced a decline of approximately 27%. Regarding race and ethnicity, Black individuals experienced more than a 20% decrease in the number of individuals with any utilization during the pandemic (approximately 23% decline in California and Michigan and approximately 26% decline in Tennessee). In California, Hispanic individuals experienced a decline of approximately 20% and in Michigan about 35%. Finally, in Michigan those who were of some other or unknown race and non-Hispanic experienced a decline in utilization of approximately 20%.

**Table 2:** Change in total ED visits before and during COVID-19 pandemic

	California			Georgia			Michigan			Tennessee		
	Before COVID	During COVID	% Difference	Before COVID	During COVID	% Difference	Before COVID	During COVID	% Difference	Before COVID	During COVID	% Difference
**Total ED Visits**	5744	4864	-15.32%	12,857	10,426	-18.91%	6064	4688	-22.69%	3507	2618	-25.35%
**Age**												
0–9	343	135	-60.64%	1623	680	-58.10%	312	142	-54.49%	329	136	-58.66%
10–19	599	493	-17.70%	1668	1141	-31.59%	323	199	-38.39%	418	343	-17.94%
20–29	2053	1784	-13.10%	3417	3289	-3.75%	1922	1606	-16.44%	1183	918	-22.40%
30–39	1627	1536	-5.59%	3114	2883	-7.42%	2039	1664	-18.39%	943	732	-22.38%
40–49	698	586	-16.05%	1953	1659	-15.05%	770	579	-24.81%	349	277	-20.63%
50–59	328	271	-17.38%	747	569	-23.83%	480	333	-30.63%	218	150	-31.19%
60+	96	59	-38.54%	335	205	-38.81%	218	165	-24.31%	67	62	-7.46%
**Sex**												
Male	2039	1,741	-14.62%	5223	3987	-23.66%	2667	2070	-22.38%	1173	884	-24.64%
Female	3705	3,123	-15.71%	7634	6439	-15.65%	3397	2618	-22.93%	2334	1734	-25.71%
**Race / Ethnicity**												
Black, non-Hispanic	5282	4,467	-15.43%				5643	4324	-23.37%	3297	2490	-24.48%
White, non-Hispanic	39	19	-51.28%				152	137	-9.87%	101	67	-33.66%
Other or Unknown Race, non-Hispanic	149	146	-2.01%				216	186	-13.89%	-	-	-
Hispanic, regardless of race	274	232	-15.33%				53	41	-22.64%	-	-	-
Other or Unknown Race/Ethnicity	-	-	-				-	-	-	109	61	-44.04%

*Note* Before COVID represents March 2019 – December 2019. During COVID represents March 2020 – December 2020. Race/ethnicity data unavailable for Georgia. Dashes represent race/ethnicity data incongruent with other states

**Table 3:** Change in number of individuals with any ED utilization before & during COVID-19 pandemic

	California			Georgia			Michigan			Tennessee		
	Before COVID	During COVID	% Difference	Before COVID	During COVID	% Difference	Before COVID	During COVID	% Difference	Before COVID	During COVID	% Difference
**All Individuals**	1191	903	-24.18%	2716	1998	-26.44%	1266	983	-22.35%	840	621	-26.07%
**Age**												
0–9	172	79	-54.07%	655	393	-40.00%	177	111	-37.29%	173	92	-46.82%
10–19	195	153	-21.54%	628	414	-34.08%	165	104	-36.97%	173	125	-27.75%
20–29	317	260	-17.98%	522	437	-16.28%	321	274	-14.64%	188	162	-13.83%
30–39	246	210	-14.63%	461	374	-18.87%	279	231	-17.20%	160	128	-20.00%
40–49	143	104	-27.27%	243	200	-17.70%	163	135	-17.18%	69	60	-13.04%
50–59	88	74	-15.91%	124	110	-11.29%	103	84	-18.45%	S	S	S
60+	30	23	-23.33%	83	70	-15.66%	58	44	-24.14%	S	S	S
**Sex**												
Male	524	388	-25.95%	1115	787	-29.42%	486	356	-26.75%	313	220	-29.71%
Female	667	515	-22.79%	1601	1211	-24.36%	780	627	-19.62%	527	401	-23.91%
**Race / Ethnicity**												
Black, non-Hispanic	1042	795	-23.70%				1166	899	-22.90%	796	591	-25.75%
White, non-Hispanic	S	S	S				39	38	-2.56%	S	S	S
Other or Unknown Race, non-Hispanic	S	S	S				44	35	-20.45%	-	-	-
Hispanic, regardless of race	82	66	-19.51%				17	11	-35.29%	-	-	-
Other or Unknown Race/Ethnicity	-	-	-				-	-	-	S	S	S

*Note* Before COVID represents March 2019 – December 2019. During COVID represents March 2020 – December 2020. Race/ethnicity data unavailable for Georgia. Dashes represent race/ethnicity data incongruent with other states. *S* represents suppressed data to comply with the data use agreement in each state

## Discussion

Across all states, reductions in ED utilization were seen both at the visit level and at the person level. Trends of recovery of ED utilization to pre-pandemic levels demonstrate similar patterns in Georgia, Michigan, and Tennessee, where a substantial initial drop is seen in the first two months of the COVID-19 pandemic, then recovery is gradual over affected months and begins to trend to near pre-pandemic levels by December 2020. The pattern of decrease and recovery is different in California, at both the visit and person level, where the initial decline is more marginal than is seen in other states and the recovery pattern is more gradual. Differences in decline in ED utilization patterns and recovery patterns between states may be driven by baseline differences in Medicaid policies and measures implemented in the context of the COVID-19 emergency intended to minimize the impact to healthcare access. In the context of the federal public health emergency, continuous enrollment for Medicaid beneficiaries was mandated, preventing renewal processes from taking place during the active emergency and preventing termination of coverage for those enrolled at the onset or gaining coverage during the course of the pandemic [28]. 

In many states, new Medicaid policies were implemented in the context of COVID-19, designed to minimize impacts to healthcare access. For example, during the pandemic, Medicaid telehealth policies were expanded in all states to include coverage of phone-only modalities, which were not available in any states pre-pandemic, and home as the originating site, which was only available in 21 states pre-pandemic [29]. In California, Medicaid coverage of telehealth pre-COVID was more extensive than in many other states. In California, telehealth services with home as the originating site and behavioral health services via telehealth were both allowed before the COVID-19 emergency [29]. This was not the case in Georgia, Michigan, or Tennessee. Such differences in policy could have lessened acute care utilization disruptions in the context of COVID-19, if Medicaid beneficiaries in California had different care-seeking patterns or were already more accustomed to seeking care through multiple modalities, pre-pandemic. Nonetheless, one study of telehealth use during the pandemic among individuals with SCD in California, Georgia, Michigan, and Tennessee noted sharp increases in telehealth utilization during April 2020 in all states [30]. The same study also found that from March 2020 through December 2020, at least one-third of individuals with SCD in Georgia, Michigan, and Tennessee utilized telehealth. In California, this number rose to nearly 80% [30]. 

Previous research studying healthcare utilization trends in the early phase of the COVID-19 pandemic has demonstrated reduced acute care utilization related to acute cardiovascular-related hospitalizations and reductions in hospital admissions resulting from ED visits, with the largest reductions seen related to non-orthopedic needs and chronic respiratory conditions [31–35]. Reported reductions in utilization were also associated with reduced average length of stay [31] and increased rate of fatality and complication [32] compared to the pre-pandemic periods, suggesting that patient perception of the COVID-19 pandemic and the risk of seeking acute care in its context may have caused patients to delay seeking attention for serious medical issues and may have resulted in care that was abbreviated when sought. A large Massachusetts study from March 11 through September 8, 2020 found a 32% reduction in hospital admissions resulting from ED visits, with the largest impact among women and children [35]. 

Previous research on acute care utilization during the COVID-19 pandemic has found large decreases in ED utilization among pediatric patients. [36] One study from January – June 2020 demonstrated declines in ED utilization as high as 74% among children under the age of 10 years in the early COVID-19 period, including a substantial decline in utilization related to serious pediatric conditions, compared to the pre-COVID period [37]. Studies of healthcare utilization among pediatric patients in the context of COVID-19 showed that pediatric viral-associated hospitalization reduced significantly compared to the pre-COVID period, possibly impacted by the implementation of masking and social distancing policies [38]. Similar to the results seen in the current study where the greatest decrease in ED utilization was consistently seen in children aged 0–9 years, ED volume began to recover through the end of the study period, but remained lower than 2019 levels across all age groups [37]. 

Prior research has also shown that observed reductions in ED visits and hospital admissions resulting from ED visits at the beginning of the pandemic were highest among those covered by publicly funded insurance [35, 37]. Most individuals with SCD are covered by public insurance, and prior research estimates that nationally more than 70% of ED visits associated with SCD are covered by Medicaid or Medicare [39]. Given the intersection of their SCD status and Medicaid coverage among the individuals in the current study, the results of our analyses are in line with prior research. At the same time, the impact may be further exacerbated for individuals with SCD who are not covered by public insurance. A recent study of healthcare costs among working age people with SCD who are covered by commercial insurance found that compared to those who do not have SCD, individuals with SCD pay four times as much out of pocket for healthcare [40]. Combined with additional uncertainty and fear surrounding the public health emergency, higher out of pocket costs associated with medical care on average, compared to the general population covered by private insurance could have further impacted individual decisions to seek acute care in the context of the COVID-19 pandemic.

This study has several limitations. This analysis assessed the utilization patterns of individuals with SCD covered by their state Medicaid programs. For this reason, this analysis is not inclusive of all individuals with SCD in each respective state and is not able to assess or account for differences in patterns of utilization between those covered by Medicaid, Medicare, and commercial insurance. Additionally, because data sources vary across states, methods used to identify ED visits were not standard across states. ED visits were identified in California and Georgia by linking each state’s study cohort with statewide hospital discharge records. This process differs from that used in Michigan and in Tennessee. ED visits were identified in Michigan using Medicaid claims among those identified in the study cohort and in Tennessee using Medicaid claims supplemented with hospital discharge records. These different methods of identifying ED visits could have yielded different identification accuracy across states. Additionally, this study did not account for additional demographic characteristics such as socio-economic status, access to healthcare, or additional comorbidities that may have influenced ED utilization patterns. Limitations notwithstanding, this study illustrates the initial impact of the COVID-19 emergency on ED utilization patterns among those with SCD, stratified by demographic factors, using a population-based surveillance system in four U.S. states.

Several key landmarks in the COVID-19 pandemic occurred after the timeframe of this study and could be objectives of further research in the future. For example, COVID-19 vaccine options were not generally available to the public during the timeframe of this study but became widely available shortly after. Disruptions in acute care utilization patterns may have been different in subsequent waves of the pandemic, in the context of general vaccine availability. Additionally, beyond December 2020, subsequent surges of infection related to the Delta and Omicron variants of COVID-19 were seen in all four states. [41] Disruptions to healthcare utilization in the context of these additional waves were likely lesser than that seen in the timeframe of the current study, as the novelty of the COVID-19 emergency situation may not have continued to be felt as strongly and, in some cases, newly circulating variants may have been perceived as having less potential to produce severe disease. Further analysis assessing the impact of these factors on acute care utilization would be useful in further understanding the disruption in acute care utilization among those suffering from SCD in the context of the COVID-19 pandemic. Future research could also build upon this study by examining the impact of delayed care on future utilization levels. We were unable to directly assess if visits to the ED by those in this study was for a current healthcare need or due to an exacerbated issue caused by previously delayed care due to the pandemic.

## Conclusions

The results of this study demonstrate how ED utilization patterns of those living with SCD were impacted by the onset of the COVID-19 pandemic. Because SCD is a condition marked by chronic complications and pain which often requires frequent medical care, those living with SCD are in an especially vulnerable position, as disruptions caused by COVID-19 have high potential to impact their quality of life. Across all four states, a decline in ED utilization during the onset of the COVID-19 emergency was seen, both at the person level and the visit level, with the most impact seen in those under the age of 10 years. By December 2020, utilization rates at both the person and visit-level were higher than their lowest observed month of April 2020, but had not fully returned to pre-COVID levels. This study confirms and extends the existing literature related to the impact of the COVID-19 emergency on healthcare utilization patterns in the US, in a unique population with increased healthcare needs, compared to the general public.

### Electronic supplementary material

Below is the link to the electronic supplementary material.

Supplementary Material 1

## Data Availability

The datasets generated and/or analyzed during the study are not publicly available because data use agreements in each state prohibit the release of the HIPAA-identifiable data utilized in this study. Aggregate level data presented in the figures and table are available upon request to the corresponding author.

## Abbreviations

ED: Emergency department
SCD: Sickle cell disease
SCDC: Sickle cell data collection program
VOC: Vaso-occlusive crisis
