# Supplementary material for: Emergency department utilization before and during the COVID-19 pandemic among individuals with sickle cell disease
Source: BMC Emerg Med. 2024 Jul 29;24:134. doi: 10.1186/s12873-024-01043-5 (PMC11287848; doi:10.1186/s12873-024-01043-5)
Supplement: Supplementary file 1 — Supplementary Material 1 [file 12873_2024_1043_MOESM1_ESM.docx]

**Supplemental Material**

To supplement the descriptive trend analysis, interrupted time series (ITS) models were estimated in each state for both the visit-level and person-level ED utilization outcome. ITS is a method of longitudinal analysis that allows examination of trends in healthcare utilization before and after a clearly defined intervention point and has been frequently used to study how the COVID-19 pandemic impacted changes in a variety of health-related outcomes.(1-3) In the current study ITS was utilized to specifically quantify the following: 1) the monthly baseline trend in ED utilization before the COVID-19 pandemic; 2) the immediate change in ED utilization following the introduction of the pandemic; and 3) the monthly trend in ED utilization following the start of the pandemic.

Drawing from studies in the broader literature that have examined overall changes in healthcare utilization before and during the pandemic in the general population (4-6), we specified a level and slope impact model.(7) Specifically, we hypothesized that the pre-pandemic trend in ED utilization would be immediately disrupted by the start of the pandemic (a level change), and that the trend in ED utilization during the pandemic would differ from the trend before the pandemic began (a slope change). For each state, we estimated the following general model:

$\ln\left( Y_{t} \right)=\beta_{0}+\beta_{1}\text{Time}+\beta_{2}X_{t}+\beta_{3}{TX}_{t}$,

where $Y_{t}$ represents the person- or visit-level ED utilization outcome at time $t$, $\beta_{0}$ represents the baseline ED utilization in January 2019, $\beta_{1}$ represents the monthly utilization slope before COVID-19, $\beta_{2}$ represents the level change in ED utilization following the pandemic, and $\beta_{3}$ represents the slope change in the monthly utilization following the start of the pandemic.

In the ITS models, a Poisson link function was utilized to account for the outcome variables representing counts and the models were offset using the log of the cohort size in each state. Seasonal variation in monthly ED utilization was accounted for using Fourier terms (pairs of sine and cosine waves). (8) Durbin-Watson statistics alongside inspection of partial autocorrelation plots and model residual plots indicated no evidence of autocorrelation in the time-series data. As described in the full study results (see main manuscript) descriptive analysis of the monthly rates warranted a one-month lag associated with the level change term in the model, therefore “starting” the impact of the pandemic in April 2024 for the purposes of the analysis. To aid in interpretation of results, model coefficients were exponentiated to represent rates of utilization.

Results of the trend analysis for the number of ED visits across all individuals with SCD are reported in Supplemental Table 1 and visually displayed in Supplemental Figure 1. At the start of the study, compared with a rate of 0, there was a significant estimated rate of ED utilization exhibited across all states ($\beta_{0}$), ranging from 250 visits/1,000 individuals in Tennessee to as high as 290 visits/1,000 individuals in Georgia. Accounting for seasonal variation in ED visits, the linear trend in visit rates before the start of the pandemic ($\beta_{1}$) was essentially flat and negligible across all states. The immediate effect of the pandemic on visit rates ($\beta_{2}$) was a statistically significant drop in estimated visits, ranging from a 10% decrease in the number of visits/1,000 individuals in California to as much as a 36% decrease in Michigan. Following the immediate decline in the visit rate and accounting for seasonal variability, Georgia, Michigan, and Tennessee experienced a significant increase in ED visits rate ranging from 2% to 3% per month beginning in April 2020 ($\beta_{3}$). Conversely, California experienced a 3% decrease in the visit rate per month beginning in April 2020.

Results of the trend analysis for the number of individuals with one or more ED visits are reported in Supplemental Table 1 and visually displayed in Supplemental Figure 2. At the start of the study, compared with a rate of 0, there was a significant estimated rate of person-level ED utilization exhibited across all states ($\beta_{0}$), ranging from 140 individuals with utilization/1,000 individuals in Michigan to as high as 170 individuals with utilization/1,000 individuals in Georgia. Accounting for seasonal variation in ED visits, the linear trend in the rate of individuals with any utilization before the start of the pandemic ($\beta_{1}$) was essentially flat and negligible across all states. The immediate effect of the pandemic on person-level visit rates ($\beta_{2}$) was a statistically significant drop in estimated person-level visits, ranging from a 20% decrease in the number of individuals with any utilization/1,000 individuals in California to as much as a 44% decrease in Michigan. Following the immediate decline in the utilization rate and accounting for seasonal variability, Georgia and Michigan experienced a significant increase in the person-level ED visit rate, indicating a 2% to 3% increase in the number of individuals with ED utilization per month beginning in April 2020 ($\beta_{3}$). Conversely, California experienced no change in monthly utilization following the initial decline, while in Tennessee a positive but not statistically significant increase of 2% per month was found.

The results of this supplemental analysis using interrupted time series demonstrated that our descriptive analysis of ED utilization trends were robust after accounting for potential seasonal variation in ED utilization among individuals with SCD. At the same time, however, we warrant strong caution in the use and causal interpretation of these results. The overall observation for this analysis (24 months total) was relatively short. This particularly limits our ability to accurately estimate the pre-pandemic seasonal trends in ED utilization, given only 15 months of pre-pandemic utilization data. We therefore encourage readers to utilize the descriptive analysis presented in the main manuscript file.

**Supplemental References**

1. Riley T, Nethery E, Chung EK, Souter V. Impact of the COVID‐19 pandemic on perinatal care and outcomes in the United States: An interrupted time series analysis. *Birth*. 2022;49(2):298-309.
2. Shah SA, Brophy S, Kennedy J, et al. Impact of first UK COVID-19 lockdown on hospital admissions: Interrupted time series study of 32 million people. *EClinicalMedicine*. 2022;49:101462.
3. Larson PS, Bergmans RS. Impact of the COVID-19 pandemic on temporal patterns of mental health and substance abuse related mortality in Michigan: an interrupted time series analysis. *Lancet Reg Health-Am*. 2022;10:100218.
4. Adjemian J, Hartnett KP, Kite-Powell A, et al. Update: COVID-19 pandemic–associated changes in emergency department visits—United States, December 2020–January 2021. *Morb Mortal Wkly Rep*. 2021;70(15):552.
5. Hartnett KP, Kite-Powell A, DeVies J, et al. Impact of the COVID-19 pandemic on emergency department visits—United States, January 1, 2019–May 30, 2020. *Morb Mortal Wkly Rep*. 2020;69(23):699.
6. Kazakova SV, Baggs J, Parra G, et al. Declines in the utilization of hospital‐based care during COVID‐19 pandemic. *J Hosp Med*. 2022;17(12):984-989.
7. Bernal JL, Cummins S, Gasparrini A. Interrupted time series regression for the evaluation of public health interventions: a tutorial. *Int J Epidemiol*. 2017;46(1):348-355.
8. Bhaskaran K, Gasparrini A, Hajat S, Smeeth L, Armstrong B. Time series regression studies in environmental epidemiology. *Int J Epidemiol*. 2013;42(4):1187-1195.

**Supplemental Table 1. Results of Interrupted Time Series Models**

|  | Model 1 - Visit Level | | |  | Model 2 - Person Level | | |
| --- | --- | --- | --- | --- | --- | --- | --- |
| Model Term | Coeff. | Rate | 95% CI |  | Coeff. | Rate | 95% CI |
| $\beta_{0}$ (Baseline Utilization) |  |  |  |  |  |  |  |
| California | -1.35*** | 0.26 | 0.25 - 0.27 |  | -1.90*** | 0.15 | 0.14 - 0.16 |
| Georgia | -1.23*** | 0.29 | 0.28 - 0.30 |  | -1.77*** | 0.17 | 0.16 - 0.18 |
| Michigan | -1.32*** | 0.27 | 0.25 - 0.28 |  | -1.93*** | 0.14 | 0.14 - 0.15 |
| Tennessee | -1.37*** | 0.25 | 0.24 - 0.27 |  | -1.81*** | 0.16 | 0.15 - 0.18 |
| $\beta_{1}$ (Before-COVID^1^ Utilization Slope) |  |  |  |  |  |  |  |
| California | 0.00 | 1.00 | 1.00 - 1.01 |  | 0.00 | 1.00 | 0.99 - 1.00 |
| Georgia | 0.00** | 1.00 | 0.99 - 1.00 |  | -0.01** | 0.99 | 0.99 - 1.00 |
| Michigan | 0.00 | 1.00 | 1.00 - 1.01 |  | 0.00 | 1.00 | 1.00 - 1.01 |
| Tennessee | -0.01* | 0.99 | 0.99 - 1.00 |  | -0.01** | 0.99 | 0.98 - 1.00 |
| $\beta_{2}$ (COVID Level Change) |  |  |  |  |  |  |  |
| California | -0.11* | 0.90 | 0.81 - 0.99 |  | -0.23*** | 0.80 | 0.69 - 0.92 |
| Georgia | -0.27*** | 0.76 | 0.71 - 0.82 |  | -0.38*** | 0.68 | 0.62 - 0.75 |
| Michigan | -0.45*** | 0.64 | 0.58 - 0.71 |  | -0.59*** | 0.56 | 0.48 - 0.64 |
| Tennessee | -0.34*** | 0.71 | 0.62 - 0.81 |  | -0.32*** | 0.73 | 0.62 - 0.86 |
| $\beta_{3}$ (Change in slope during COVID^2^) |  |  |  |  |  |  |  |
| California | -0.03*** | 0.97 | 0.96 - 0.99 |  | 0.00 | 1.00 | 0.98 - 1.02 |
| Georgia | 0.02*** | 1.02 | 1.01 - 1.03 |  | 0.02** | 1.02 | 1.00 - 1.03 |
| Michigan | 0.03*** | 1.03 | 1.02 - 1.05 |  | 0.03** | 1.03 | 1.01 - 1.05 |
| Tennessee | 0.03** | 1.03 | 1.01 - 1.05 |  | 0.02 | 1.02 | 0.99 - 1.05 |

Note: all models control for seasonal variability. * *p* $\leq$ .05, ** *p* $\leq$ .01, *** *p* $\leq$ .001.

1. Before COVID timeline = January 2019 through February 2020

2. During COVID timeline = March 2020 through December 2020 (with one-month lag)

**
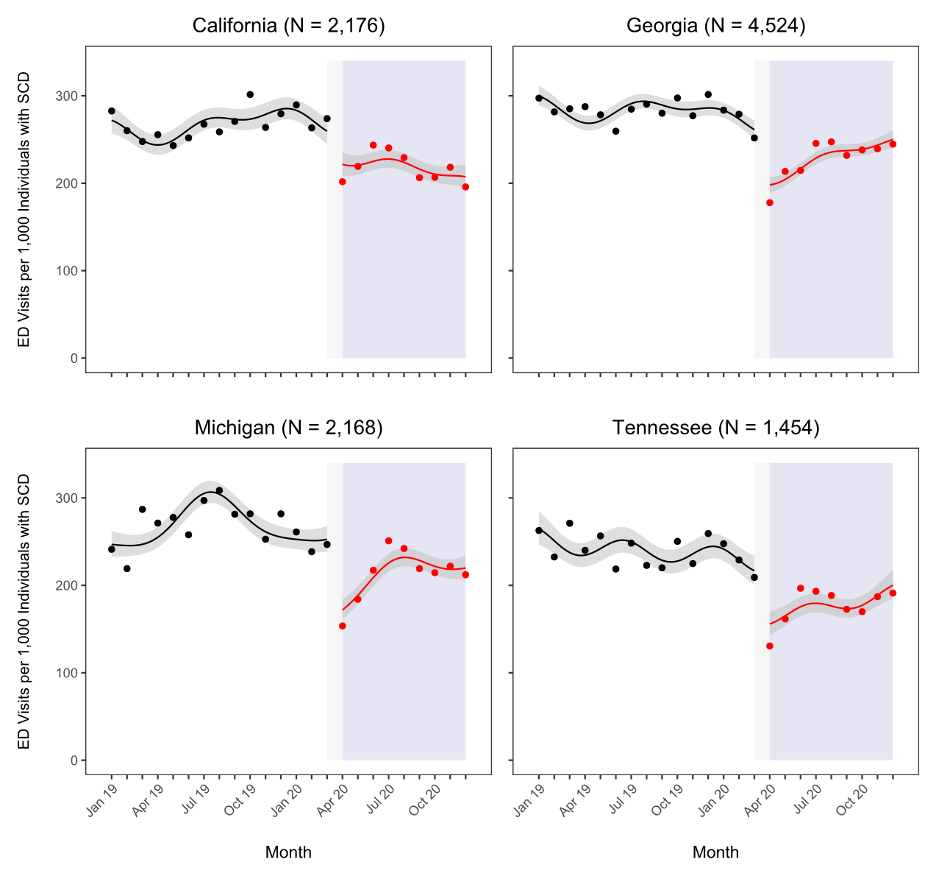
**

**Supplemental Figure 1. Monthly Trends in Emergency Department Visits per 1,000 Individuals with Sickle Cell Disease.** Black and red points represent the observed rate during the pre-pandemic and pandemic periods, respectively. Solid black lines represent the model-predicted rate before the start of the pandemic, solid red lines represent the model-predicted rate during the pandemic, and grey bands represent 95% confidence intervals. The grey shaded region represents the one-month lag associated with the model level change term. The purple shaded region represents the pandemic period.

**
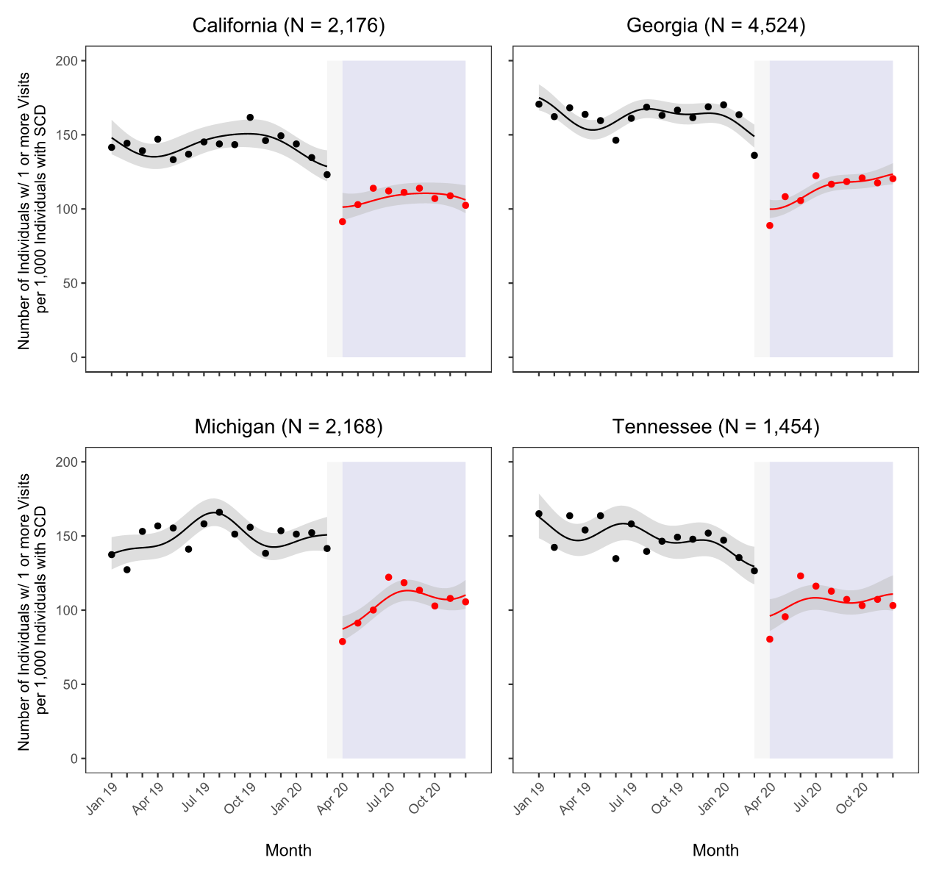
**

**Supplemental Figure 2. Monthly Trends in the Number of Individuals with One or More Emergency Department Visits, per 1,000 Individuals with Sickle Cell Disease.** Black and red points represent the observed rate during the pre-pandemic and pandemic periods, respectively. Solid black lines represent the model-predicted rate before the start of the pandemic, solid red lines represent the model-predicted rate during the pandemic, and grey bands represent 95% confidence intervals. The grey shaded region represents the one-month lag associated with the model level change term. The purple shaded region represents the pandemic period.
